# Id2 exacerbates the development of rheumatoid arthritis by increasing IFN‐γ production in CD4^+^ T cells

**DOI:** 10.1002/ctm2.70242

**Published:** 2025-03-06

**Authors:** Haoyang Sun, Jinlin Miao, Kui Zhang, Peiyan Zhang, Haomiao Shen, Jiawei Wang, Bei Zhang, Junfeng Jia, Zhaohui Zheng, Ping Zhu

**Affiliations:** ^1^ Department of Clinical Immunology Xijing Hospital, Fourth Military Medical University Xi'an China; ^2^ Department of Cell Biology, National Translational Science Center for Molecular Medicine Fourth Military Medical University Xi'an China

**Keywords:** abatacept, IFN‐γ, inhibitor of differentiation 2, rheumatoid arthritis, T cell

## Abstract

**Purpose:**

This research investigates the role of inhibitor of differentiation 2 (Id2) in the synthesis of pro‐inflammatory cytokines, specifically interferon‐γ (IFN‐γ) and interleukin‐17 (IL‐17), by various subsets of T cells, and its pathogenic role in rheumatoid arthritis (RA).

**Methods:**

Flow cytometry was employed to assess T‐cell activation and Id2 expression in 72 RA patients and 23 healthy controls. In vitro, peripheral blood mononuclear cells were treated with either an Id2 inhibitor or a T‐cell co‐stimulation inhibitor. An in vivo collagen‐induced arthritis (CIA) model was established using T‐cell‐specific Id2 knockout mice. Additionally, follow‐up observations were conducted among treated RA patients.

**Results:**

T‐cell activation levels in RA synovial fluid were significantly elevated. A positive correlation was found between increased IFN‐γ and Id2 expression. In vitro, antagonising Id2 reduced IFN‐γ production after T‐cell activation. T‐cell‐specific Id2 knockout mice exhibited a diminished occurrence and severity of CIA, along with a significant decrease in IFN‐γ expression. Clinical monitoring indicated that Id2‐induced circulating T‐cell IFN‐γ expression significantly decreased following treatment with the T‐cell activation inhibitor abatacept.

**Conclusion:**

The data suggest that high Id2 expression is a critical regulator of pro‐inflammatory cytokine upregulation, particularly IFN‐γ, by hyperactivated T cells in RA, potentially exacerbating the disease. Inhibiting Id2 expression or function may offer new therapeutic approaches for RA joint inflammation.

**Key points:**

- Pro‐inflammatory cytokines are significantly upregulated in the synovial fluid T cells in rheumatoid arthritis patients.
- The expression of pro‐inflammatory cytokine interferon‐γ (IFN‐γ) positively correlates with the high expression of inhibitor of differentiation 2 (Id2).
- The inhibition or ablation of Id2 can effectively suppress IFN‐γ production and the onset and progression of arthritis.

## Graphical abstract

Schematic mechanism of inhibitor of differentiation 2 (Id2)‐mediated interferon‐γ (IFN‐γ) expression upregulation in the pathogenesis of rheumatoid arthritis (RA).

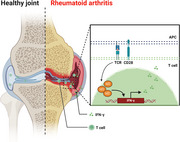

## 1 INTRODUCTION

Rheumatoid arthritis (RA) is a persistent autoimmune disorder distinguished by systemic polyarthritis and joint destruction.^1^, ^2^, ^3^ The pathogenesis, however, remains incompletely understood. The typical pathology is characterised by synovitis, which is triggered by the abnormal accumulation and interactive activation of various immune cells, particularly autoreactive T cells.^4^, ^5^, ^6^ Notably, hyperactivated autoreactive T cells in inflamed joints, particularly interferon‐γ (IFN‐γ)‐expressing T helper type 1 (Th1) and interleukin‐17 (IL‐17)‐expressing Th17, interact with macrophages, B cells and fibroblast‐like synoviocytes to mediate the production of autoantibodies and the degradation of cartilage.^7^, ^8^, ^9^ The pro‐inflammatory cytokines produced by these autoreactive T cells have been associated with the onset and perpetuation of various autoimmune disorders, including RA.^10^, ^11^, ^12^ Prior research has established that IFN‐γ and IL‐17 are substantially elevated in RA peripheral blood (PB) and synovial fluid (SF),^13^, ^14^ and this is further supported by evidence that cytokines expression was diminished following treatment and disease remission.^15^, ^16^ A growing number of targeted drugs, such as abatacept (ABA), which blocks T‐cell co‐stimulatory signalling, and tumour necrosis factor inhibitors (TNFi), which antagonise TNF‐α, have been demonstrated to suppress the synthesis of cytokines for the treatment of RA.^17^, ^18^ However, the mechanisms underlying the disproportionate number of pro‐inflammatory T cells and their aberrant cytokine production in RA remain unclear.

Previous studies have shown that the inhibitor of differentiation 2 (Id2), a natural repressor of E‐protein transcription factors with a helix‒loop‒helix (HLH) structure,^19^ is widely distributed in various immune cells and undergoes significant changes during the activation, proliferation, differentiation, cytokine secretion and apoptosis of T cells.^20^, ^21^, ^22^ Recent studies have also implicated Id2 in the aetiology of immunological disorders. In animal models, Id2 deficiency restricts the expansion of the effector T cell and impairs its capacity to produce pro‐inflammatory cytokines, ultimately hindering the initiation of experimental autoimmune encephalomyelitis (EAE).^23^ In addition, clinical studies have shown that Id2 expression is reduced in the colonic tissues of ulcerative colitis, which helps to maintain the barrier function of the intestinal mucosa by decreasing macrophage and neutrophil infiltration, as well as the expression of inflammatory factors.^24^ Strikingly, Id2 expression is markedly downregulated in the inflamed intestinal mucosa and circulating CD4^+^ T cells of inflammatory bowel disease (IBD).^25^ Our prior exploration also revealed that Id2 is differentially expressed in different T cell subsets in RA.^26^ However, the pathophysiological mechanisms underlying Id2's involvement in modulating cytokine expression, which exert a determinative function in the aetiology of RA joint inflammation, remain incompletely understood.

This research examined the potential effects of Id2 on pro‐inflammatory cytokine expression in RA T cells, offering new perspectives for regulating the pro‐inflammatory function of T cells and developing targeted therapies.

## 2 METHODS

### 2.1 Subjects

This research recruited 72 active RA patients (12 males and 60 females; mean age 48.63 ± 13.79 years) adhered to the 2010 ACR/EULAR RA classification criterion^27^ from Xijing Hospital, from September 2020 to July 2022. Patients with other inflammatory diseases or who had received high‐dose corticosteroids (such as prednisone exceeding 10 mg/day) and any biologic disease‐modifying antirheumatic drugs within the past 3 months were excluded. SF was collected from 17 RA patients. Additionally, 23 healthy controls (HCs) without a family history or other known chronic illnesses were included in this study (five males and 18 females; mean age 45.91 ± 11.27 years). These subjects comprised cohort 1. Clinical data and laboratory test indicators were recorded. Laboratory data and clinical features were collected simultaneously during sampling (Table 1).

**TABLE 1:** Demographic and clinical characteristic of subjects in cohort 1.

	RA (*n* = 72)	HC (*n* = 23)
Parameter		
Age (years)	44.0 (36.0‒56.0)	48.5 (41.0‒57.75)
Gender (F/M)	60/12	18/5
Duration of disease (months)	60.0 (12.0‒117.0)	NA
ACPA+, *n* (%)	40 (55.56)	NA
RF+, *n* (%)	57 (79.17)	NA
Tender joint count/28	7.5 (3.0‒12.0)	NA
Swollen joint count /28	3.0 (1.25‒6.0)	NA
ESR (mm/h)	23.0 (8.0‒42.5)	NA
CRP (mg/dL)	1.04 (.30‒2.39)	NA
DAS28‐CRP	4.77 ± 1.19	NA
Medication		
Corticosteroids use, *n* (%)	4 (5.6)	NA
MTX use, *n* (%)	16 (22.2)	NA
LEF use, *n* (%)	19 (26.4)	NA
HCQ use, *n* (%)	14 (19.4)	NA
SZZ use, *n* (%)	10 (13.9)	NA

*Note*: Data are presented as numbers (percentage), mean ± standard deviation or median (interquartile range).

Abbreviations: ACPA, anti‐cyclic citrullinated peptide antibody; CRP, C‐reactive protein; DAS28‐CRP, 28‐joint disease activity score based on CRP; ESR, erythrocyte sedimentation rate; HC, healthy control; HCQ, hydroxychloroquine; LEF, leflunomide; MTX, methotrexate; NA, not applicable; RA, rheumatoid arthritis; RF, rheumatoid factor; SZZ, sulphasalazine.

Furthermore, 51 patients who had received either ABA or TNFi for a minimum of 3 months and had completed the follow‐up period included in the 72 RA patients were categorised into ABA group (*n* = 25) and TNFi group (*n* = 26; adalimumab 14, etanercept 12). These 51 RA patients comprised cohort 2. Disease‐modifying antirheumatic drugs, typified by sulphasalazine, leflunomide, hydroxychloroquine and methotrexate, may be prescribed depending on the patient's individual circumstances (Table 2). All drugs were administered in accordance with the recommended dosage and route of administration. At baseline, no notable differences were observed among the groups concerning demographic characteristics, clinical data and laboratory test parameters. This research received approval from the Ethics Committee of Xijing Hospital (KY20192006‐F‐1), and informed consent was obtained.

**TABLE 2:** Clinical and biological parameters of patients in cohort 2.

	ABA group (*n* = 25)	TNFi group (*n* = 26)
Parameter		
Age (years)	47.48 ± 13.61	51.46 ± 14.42
Gender (F/M)	19/6	21/5
Disease duration (months)	24.0 (6.0‒138.0)	60.0 (11.0‒126.0)
ACPA+, *n* (%)	16 (64.0)	12 (46.2)
RF+, *n* (%)	21 (84.0)	23 (88.5)
ESR (mm/h)	29.0 (6.8‒64.3)	26.0 (10.0‒53.3)
CRP (mg/dL)	1.5 (.2‒5.2)	.7 (.3‒3.8)
DAS28‐CRP	5.06 ± 1.53	4.84 ± .96
Medication	DMARDs + ABA	DMARDs + TNFi
Corticosteroids use, *n* (%)	3 (12.0)	5 (19.2)
MTX use, *n* (%)	23 (92.0)	23 (88.5)
LEF use, *n* (%)	21 (84.0)	22 (84.6)
HCQ use, *n* (%)	12 (48.0)	10 (38.5)
SZZ use, *n* (%)	10 (40.0)	7 (26.9)

*Note*: Data are presented as numbers (percentage), mean ± standard deviation or median (interquartile range).

Abbreviations: ABA, abatacept; ACPA, anti‐cyclic citrullinated peptide antibody; CRP, C‐reactive protein; DAS28‐CRP, 28‐joint disease activity score based on CRP; DMARDs, disease‐modifying antirheumatic drugs; ESR, erythrocyte sedimentation rate; HCQ, hydroxychloroquine; LEF, leflunomide; MTX, methotrexate; RF, rheumatoid factor; SZZ, sulphasalazine; TNFi, tumour necrosis factor inhibitors.

### 2.2 Flow cytometry analysis

The subsequent antibodies were utilised: phycoerythrin (PE) anti‐human CD4 (clone: RPA‐T4), fluorescein isothiocyanate (FITC) anti‐human CD8 (clone: RPA‐T8), brilliant violet (BV) 711 anti‐human CD69 (clone: FN50), BV421 anti‐human CD25 (clone: BC96), BV711 anti‐human IFN‐γ (clone: 4S.B3), allophycocyanin anti‐human IL‐17 (clone: BL168), peridinin chlorophyll protein (PerCP)/Cyanine5.5 anti‐mouse CD4 (clone: RM4‐5), FITC anti‐mouse CD8 (clone: 53–6.7), BV711 anti‐mouse IFN‐γ (clone: XMG1.2), PE anti‐mouse IL‐17 (clone: TC11‐18H10.1) (all from Biolegend) and eFluor 450 anti‐human Id2 (clone: ILCID2) (eBiosciences). For intracellular and intranuclear factor staining, PB and SF samples were incubated with Cell Activation Cocktail, PMA/Ionomycin (Biolegend) for 4 h before analysis. After 20 min of surface staining in the dark, erythrocytes were lysed. Intracellular cytokines and transcription factors were subsequently stained using the corresponding antibodies and the Foxp3 Staining Buffer Set (eBiosciences). The stained samples were resuspended for analysis via flow cytometry (Beckman Coulter Inc.). To ascertain the comparability of mean fluorescence intensity (MFI) across various samples, a standardised protocol was meticulously adhered to for sample processing and staining. Consistent voltage and threshold parameters, as well as uniform gating strategies, were systematically applied throughout the experimental procedure. In the comparison of the MFI of samples from various patient groups and tissue sources, the MFI were standardised by deducting the MFI of unstained samples from those of the experimental samples.

### 2.3 Cell culture

Peripheral blood mononuclear cells (PBMCs) were separated and resuspended at a density of 5 × 10^6^ cells/mL with RPMI medium complemented with 10% foetal bovine serum. The cell suspension was distributed in a 96‐well plate, achieving a final volume of 200 µL. Cell samples activated with anti‐CD3 (.25 µg/mL) and anti‐CD28 (.25 µg/mL) antibodies were treated with helichrysetin (5 µg/mL, Topscience), an selective binding inhibitor of Id2, for 48 h. Samples were harvested to assess the expression of Id2 and cytokines using flow cytometry.

### 2.4 Mice

Id2^fl/fl^ mice and Cd4^cre^ mice were generously provided by Professor Yuzhang Wu of the Third Military Medical University. Id2^fl/fl^ Cd4‐Cre^−^ (wild type [WT]) and Id2^fl/fl^ Cd4‐Cre^+^ (knockout [KO]) mice produced through the breeding of Id2^fl/fl^ mice with Cd4^cre^ mice. The mice were maintained in a controlled environment that was free from specific pathogens and controlled temperature and humidity and a regular circadian rhythm. Sex‐matched mice were randomly assigned to groups for subsequent experiments at 10–12 weeks of age. The animal experiments received approval from the Ethics Committee of the National Translational Science Center for Molecular Medicine (2021‐NTSCMM‐ID015).

### 2.5 Collagen‐induced arthritis model

On day ‒21, mice were administered with 200 µg of chicken type II collagen (Chondrex) dispersed in complete Freund's adjuvant (Chondrex), followed by another administered of the same concentration of collagen dispersed in incomplete Freund's adjuvant (Chondrex) on day 0, according to the protocol.^28^ The degree of redness and swelling in the interphalangeal, metacarpophalangeal, carpal and tarsal joints of all paws was assessed using a grading scale that ranges from 0 to 4. Score 0 indicates normality. Scores of 1, 2, 3 and 4 indicate redness and swelling in one, two, three and the entire paw without clear anatomical definition, respectively.^29^

### 2.6 Statistical analyses

Data were examined via GraphPad Prism software (version 8.3). Normally distributed data are exhibited as means ± standard deviations, while skewed data are exhibited as median (interquartile range). For normally distributed data with equal variances, Student's *t*‐test and one‐way analysis of variance were employed to evaluate differences among groups, respectively. Bonferroni's test was used for multiple comparisons. Mixed‐effect analysis is employed in the presence of missing values. For skewed data or variance heterogeneity, the Mann‒Whitney *U*‐test, Wilcoxon's test or Kruskal‒Wallis *H*‐test were utilised, with Dunn's test applied for multiple comparisons. Spearman's correlation test was applied for evaluating the degree of correlation. *p* < .05 was deemed statistically significant.

## 3 RESULTS

### 3.1 Increased T‐cell activation and IFN‐γ expression in the synovial fluid of patients with active RA

We first assessed T‐cell activation markers CD25 and CD69 in the PB and SF of patients with active RA and HCs (Figure S1a,b). The proportion of CD25^+^ CD8^+^ T cells was higher in RA PB compared to HC PB (Figure S1c). However, no statistically significant difference was detected in the proportion of CD25^+^ CD4^+^ T cells between the groups (Figure S1c). The proportions of CD69^+^ CD4^+^ and CD69^+^ CD8^+^ T cells in RA SF notable increased (Figure S1d). Further pairwise comparisons of PB and SF from the same patients revealed no difference in CD25^+^ T cells (Figure S1e). However, consistent with previous findings,^30^ CD69^+^ T cells were abundant in RA SF (Figure S1f). We subsequently analysed the proportions of pro‐inflammatory T‐cell subgroups (Figure S2a) and found that the proportion of IFN‐γ^+^ CD8^+^ T cells in SF was lower than that in RA PB. Additionally, IL‐17^+^ CD8^+^ T cells in SF elevated when relative to HCs (Figure S2b). We then compared the quantity of IFN‐γ or IL‐17 expressed by T cells and found that IFN‐γ production (MFI) was dramatically elevated in CD4^+^ SF T cells in RA relative to RA PB (Figure S2c).

### 3.2 Increased IFN‐γ expression in T cells is positively correlated with Id2

**FIGURE 1:**
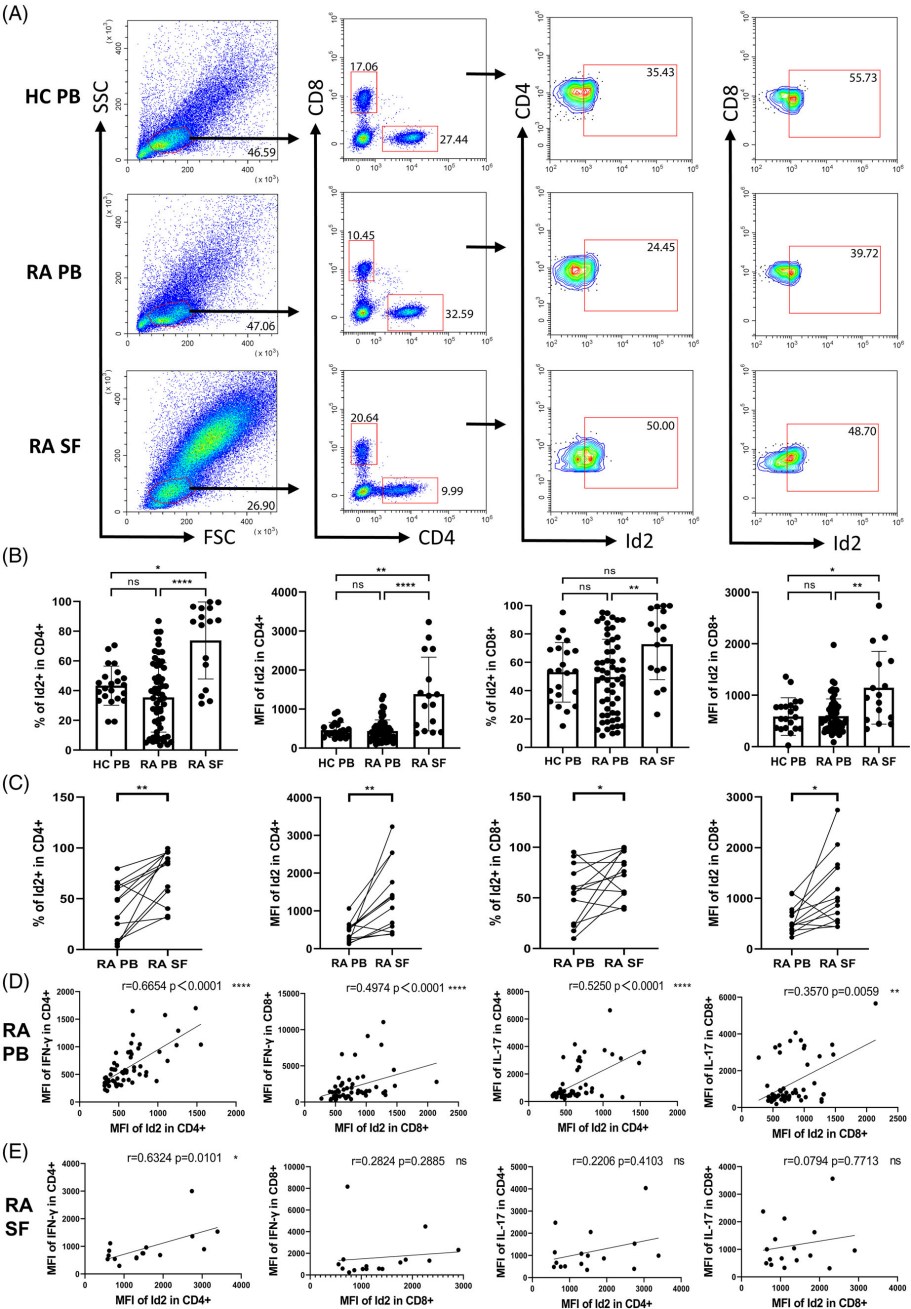
Increased interferon‐γ (IFN‐γ) expression was correlated with inhibitor of differentiation 2 (Id2) level in rheumatoid arthritis (RA) patients. (A) Representative flow cytometric plots depicting Id2 expression in T cells. (B) Percentages of Id2^+^ in CD4^+^ and CD8^+^ T cells and corresponding mean fluorescence intensity (MFI). (C) Paired comparison of Id2^+^ in CD4^+^ and CD8^+^ T cells and MFI in peripheral blood (PB) and synovial fluid (SF) from the same patient. (d and e) Correlation between Id2 (MFI) and interferon‐γ (IFN‐γ) (MFI) or interleukin‐17 (IL‐17) (MFI) from RA PB and SF. Data are from 22 healthy controls (HCs), 68 RA patients and 16 RA SF samples that were processed for stimulation prior to flow staining. Kruskal‒Wallis *H*‐test followed by Dunn's test (B), Wilcoxon's test (C) and Spearman's correlation test (D and E) were used. ns: not significant; ^*^ *p* < .05; ^**^ *p* < .01; ^***^ *p* < .001; ^****^ *p* < .0001.

Given the prominent contribution of Id2 to the development and functional regulation of the T lymphocyte lineage, we next evaluated the expression of Id2 and revealed that Id2 was highly expressed in hyperactivated synovial T cells relative to RA PB and HC PB (Figure 1A,B). Paired *t*‐tests confirmed that Id2 expression in SF T cells was higher than in PB (Figure 1C). Additionally, correlation analysis only revealed that Id2 in CD4^+^ T cells exhibited a significant positive correlation with IFN‐γ exclusively in RA PB (Figure 1D). Similarly, in the RA SF samples, Id2 positively correlates with IFN‐γ was also demonstrated in CD4^+^ T cells, but no significant correlation was detected in other outcomes (Figure 1E).

### 3.3 Increased Id2 and IFN‐γ expression after T‐cell activation in vitro

**FIGURE 2:**
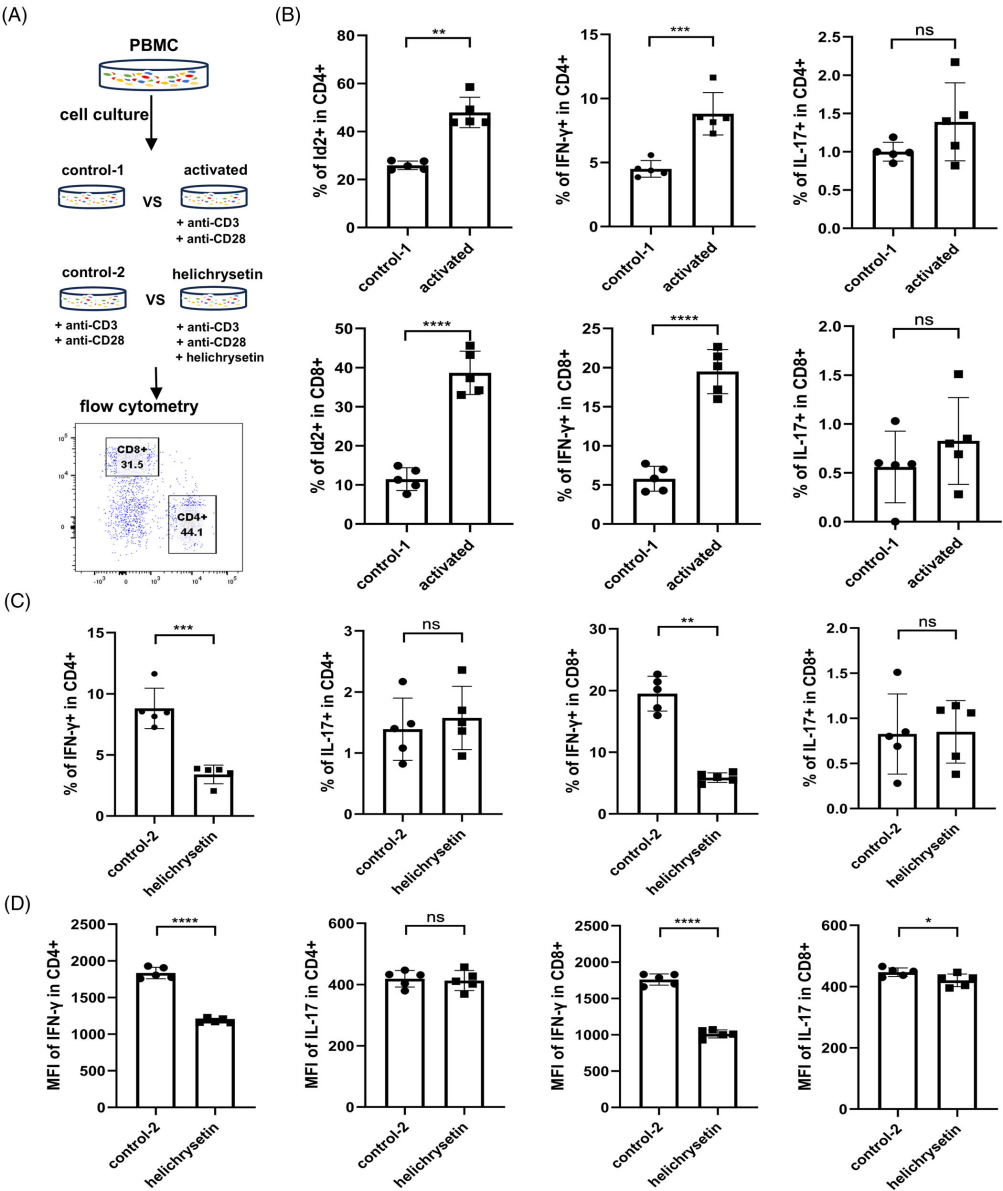
Increased inhibitor of differentiation 2 (Id2) and interferon‐γ (IFN‐γ) expression after T‐cell activation in vitro. (A) In vitro treatment scheme for T cells expression of Id2 and pro‐inflammatory cytokines. (B) Comparison of Id2^+^, IFN‐γ^+^ and IL‐17^+^ T cells in peripheral blood of healthy controls (HCs) before and after 48 h of anti‐CD3 (.25 µg/mL) and anti‐CD28 (.25 µg/mL) activation. (C and D) Production of IFN‐γ and interleukin‐17 (IL‐17) by CD4^+^ and CD8^+^ T cells from HCs treated with helichrysetin (5 µg/mL) for 48 h with anti‐CD3 and anti‐CD28 combined activation. Data are from five independent experiments. All samples were processed for stimulation prior to flow cytometry staining. Student's *t*‐test (B‒D) was used. ns: not significant; ^*^ *p* < .05; ^**^ *p* < .01; ^***^ *p* < .001; ^****^ *p* < .0001.

To examine the function of Id2, PBMCs from HCs were isolated and activated to evaluate the expression of Id2, as well as IFN‐γ and IL‐17 (Figure 2A). As anticipated, T‐cell activation yielded a notable elevation in the proportions of Id2^+^ and IFN‐γ^+^ T cells (Figures S3a and 2b). Previous studies have demonstrated that helichrysetin, extracted from Helichrysum odoratissimum, can antagonise Id2 protein, preventing its binding to other HLH proteins and exerting negative regulatory effects.^31^ To investigate the regulatory relationship between Id2 and pro‐inflammatory cytokines, we compared IFN‐γ and IL‐17 expression after activation in the presence or absence of the Id2 inhibitor, helichrysetin. The results indicated that antagonizing Id2 resulted in a notable reduction in the proportions of IFN‐γ‐expressing T cells, but the proportion of IL‐17‐expressing T cells did not change significantly (Figures S3b and 2c). MFI detection also revealed a significant decrease in IFN‐γ following Id2 blockade. Meanwhile, IL‐17 production in CD8^+^ T cells was slightly decreased but remained unaffected in CD4^+^ T cells (Figure 2D).

### 3.4 T‐cell‐specific knockout Id2 inhibits the development of collagen‐induced arthritis

**FIGURE 3:**
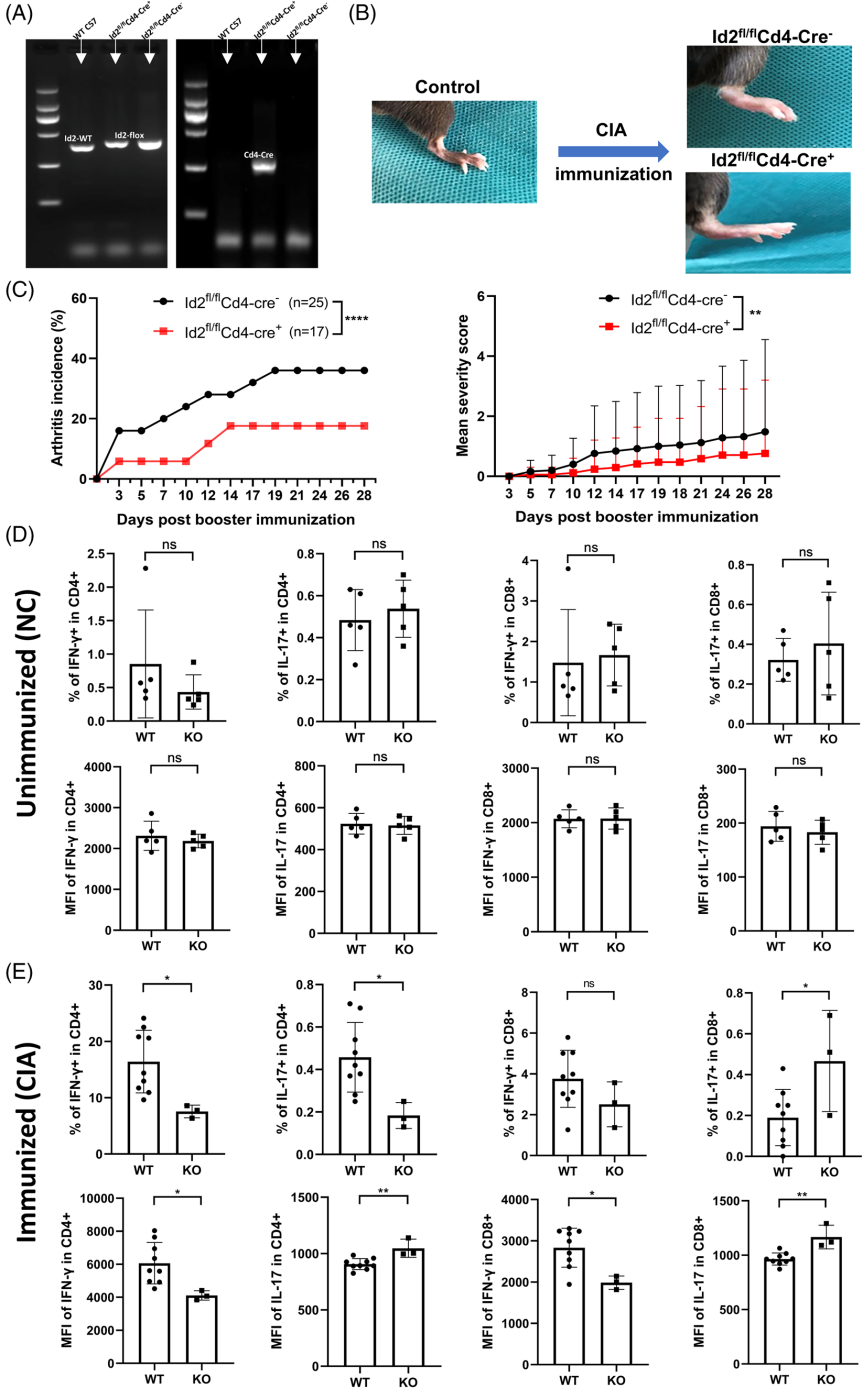
T‐cell‐specific knockout (KO) inhibitor of differentiation 2 (Id2) inhibits collagen‐induced arthritis (CIA) development. (A) The panels show PCR genotyping of alleles. The left panel amplifies the Id2‐WT and Id2‐flox cDNA, while the right panel amplifies the Cd4‐Cre cDNA. (B) Representative images of hind limb swelling before and after CIA immunisation. (C) Dynamic changes in arthritis incidence and clinical arthritis scores following CIA immunisation. (D) Presentations of interferon‐γ (IFN‐γ) and interleukin‐17 (IL‐17) in the spleen of unimmunised Id2^fl/fl^ Cd4‐Cre^−^ mice (wild type [WT], *n* = 5) and Id2^fl/fl^ Cd4‐Cre^+^ mice (KO, *n* = 5). (E) Presentations of IFN‐γ and IL‐17 in the spleen of immunised Id2^fl/fl^ Cd4‐Cre^−^ mice (WT, *n* = 9) and Id2^fl/fl^ Cd4‐Cre^+^ mice (KO, *n* = 3) with CIA. All samples were processed for stimulation prior to flow staining. Bonferroni's test (c) and Student's *t*‐test (d and e) were used. ns: not significant; ^*^ *p* < .05; ^**^ *p* < .01; ^***^ *p* < .001; ^****^ *p* < .0001.

To further examine the function of Id2 in vivo, Id2^fl/fl^ Cd4‐Cre^+^ mice (KO) with Id2 KO in T cells were generated, and Id2^fl/fl^ Cd4‐Cre^−^ mice served as WT controls (Figure 3A). These mice and their littermates were used to establish a collagen‐induced arthritis (CIA) model to observe the effect of Id2 deletion in T cells on the incidence and severity of arthritis (Figure 3B). Compared to WT mice, KO mice displayed a lower incidence and severity of clinical arthritis (Figure 3C). Additionally, we analysed the quantities of pro‐inflammatory T cells and their capacity to synthesise pro‐inflammatory cytokines in the presence of CIA immunisation. Under non‐immunised conditions, splenic T cells derived from WT and KO mice exhibited comparable IFN‐γ or IL‐17 production (Figures S4a and 3d). Following immunisation, the percentage of IFN‐γ‐expressing splenic CD4^+^ T cells and the MFI of IFN‐γ in splenic CD4^+^/8^+^ T cells were lower in KO mice with limb inflammation compared to those in WT mice. However, there was inconsistency in the percentage and MFI of IL‐17‐expressing T‐cell subsets when comparing the two groups (Figures S4b and 3e).

### 3.5 Inhibition of T‐cell activation decreases Id2 and IFN‐γ expression in RA patients

**FIGURE 4:**
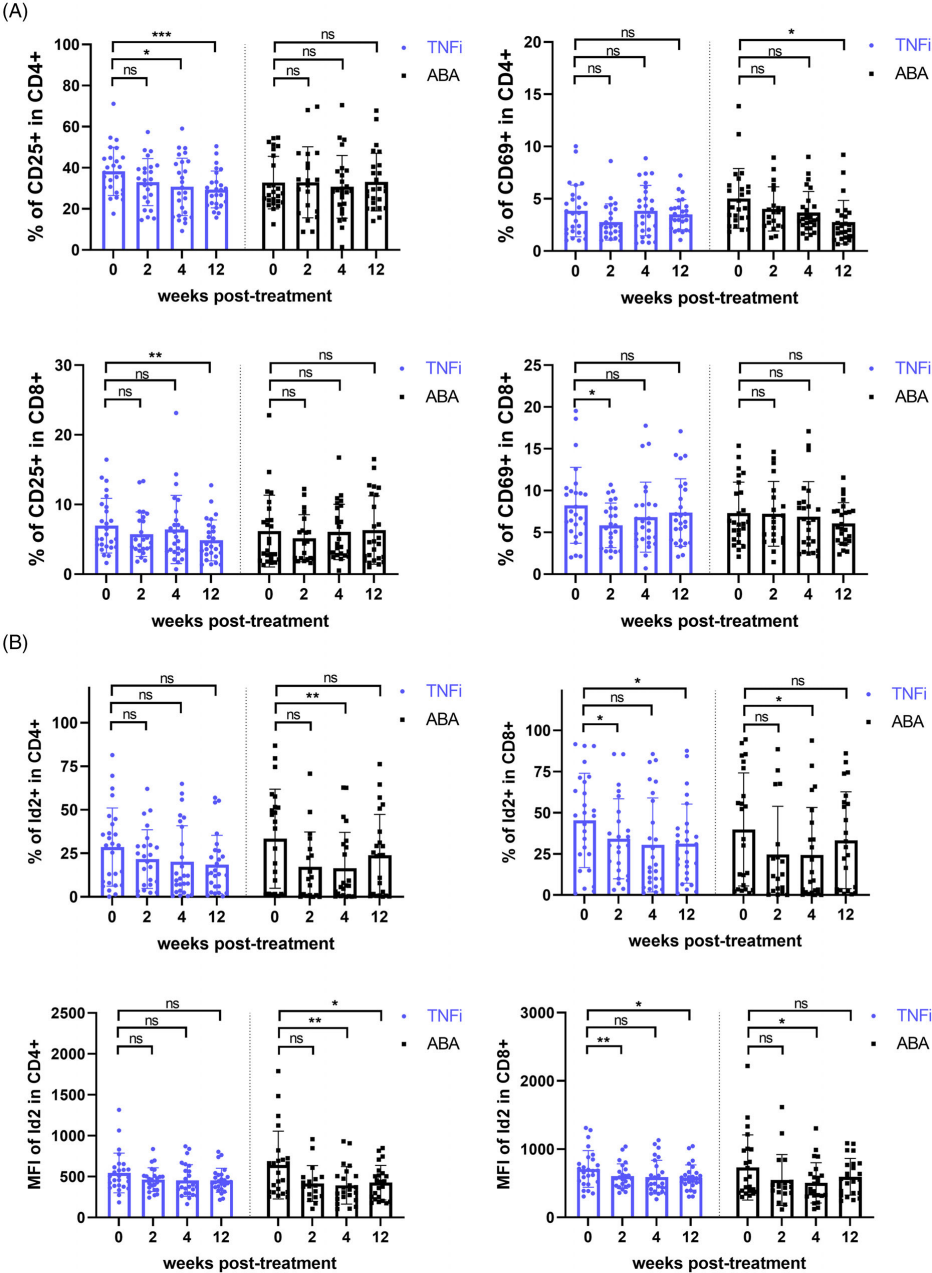
Repression of T‐cell activation decreases inhibitor of differentiation 2 (Id2) expression in rheumatoid arthritis (RA) patients. (A) CD25 and CD69 in circulating T cells of RA patients treated with tumour necrosis factor inhibitors (TNFi) and abatacept (ABA) at different time points. (B) Levels of Id2 in circulating T cells of RA patients treated with TNFi and ABA at different time points. Samples were processed for stimulation prior to flow staining. Data are from 51 patients. Mixed‐effect analysis followed by Dunn's test was used. ns: not significant; ^*^ *p* < .05; ^**^ *p* < .01; ^***^ *p* < .001.

**FIGURE 5:**
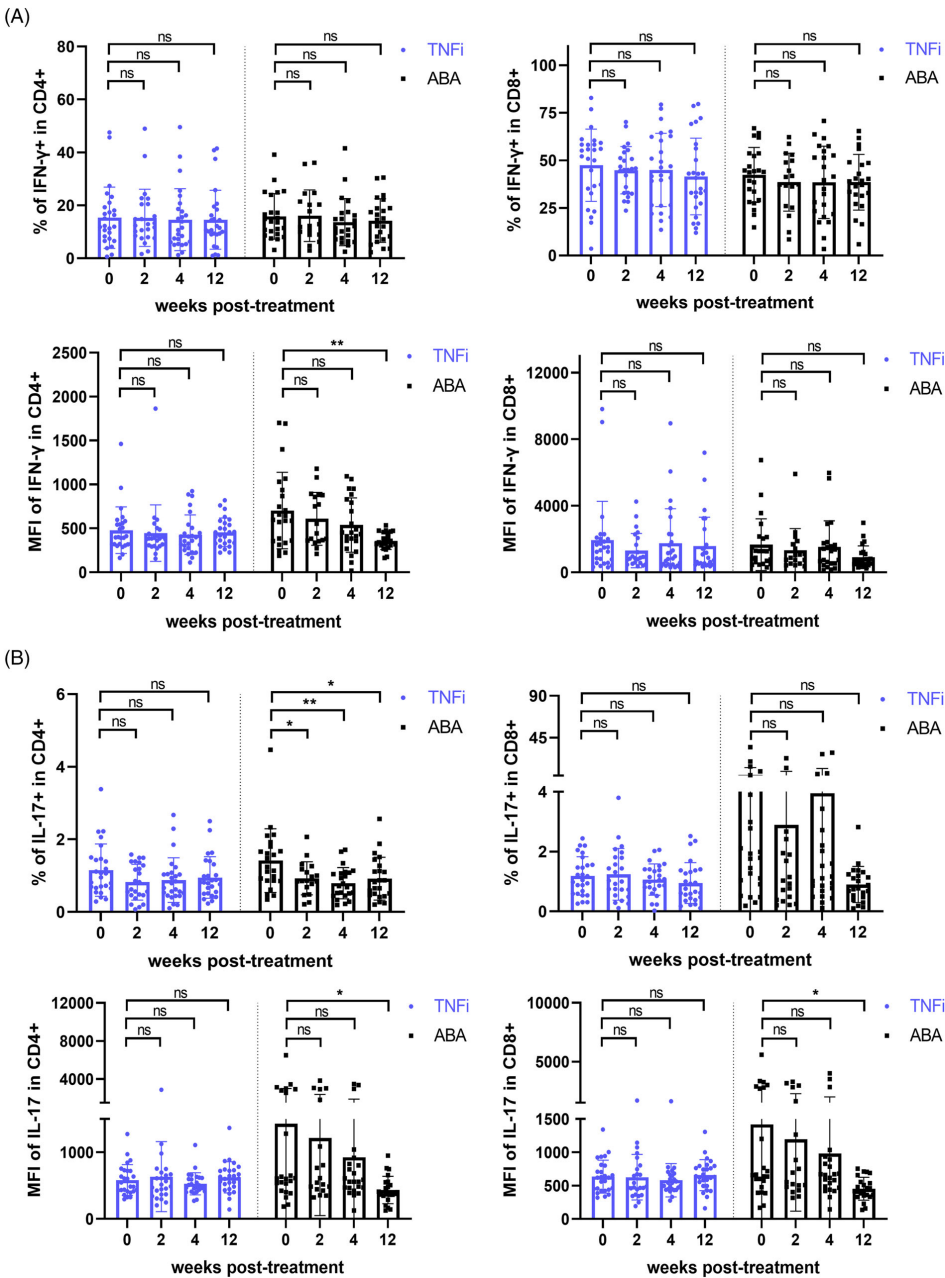
Repression of T‐cell activation decreases interferon‐γ (IFN‐γ) expression in rheumatoid arthritis (RA) patients. (A and B) Levels of IFN‐γ and interleukin‐17 (IL‐17) in circulating T cells of RA patients treated with tumour necrosis factor inhibitors (TNFi) and abatacept (ABA) at different time points. Samples were processed for stimulation prior to flow staining. Data are from 51 patients. Mixed‐effect analysis followed by Dunn's test was used. ns: not significant; ^*^ *p* < .05; ^**^ *p* < .01.

To further assess the impact of activation inhibition on Id2 and cytokine secretion in RA patients, we divided 51 patients into the TNFi group (*n* = 26) and ABA group (*n* = 25) based on their treatment protocols and monitored them during the 12‐week treatment period. Similar to previous clinical trials of ABA, CD69^+^ CD4^+^ T cells within the ABA group decreased over the treatment duration, with a statistically significant difference at the 12‐week mark. After continuous treatment, a notable decline in CD25^+^ CD4^+^ T and CD25^+^ CD8^+^ T was also evident in TNFi group (Figure 4A). Furthermore, consistent with this, Id2 was markedly diminished in ABA group at both 4 and 12 weeks after treatment (Figures S5a and 4b). Upon further examination, IFN‐γ and IL‐17 synthesised by CD4^+^ T cells were notably diminished in ABA group, consistent with previous results (Figure 5A,B).

## 4 DISCUSSION

Research has demonstrated that the abnormal activation, proliferation and differentiation of T cells are significant factors contributing to the development of RA.^6^, ^32^ Throughout all stages of RA, various inflammatory cells, especially autoreactive T cells, become activated and secrete numerous pro‐inflammatory factors, leading to joint synovitis, inflammation, joint pain and swelling.^32^ During this process, the levels of Th1 and Th17 increase, and cytokines secretion elevate, which is a critical factor in the instigation and exacerbation of local inflammation in RA.^33^, ^34^, ^35^ It has been proven that inhibiting the formation of pro‐inflammatory T‐cell subsets or antagonising excessive pro‐inflammatory cytokines constitutes an optimal strategy for alleviating RA.

In this research, we first observed that T cells demonstrated substantial activation in the inflamed joints of RA compared to RA PB and HC PB. Given that the overactivation of T cells at inflammation sites is directly related to the generation of pro‐inflammatory subsets, we further analysed the T‐cell subsets expressing the principal cytokines, IFN‐γ and IL‐17. Despite the absence of a significant rise in the proportion of IFN‐γ‐ or IL‐17‐expressing T cells in patients, we demonstrate that CD4^+^ T cells secreted significantly more IFN‐γ in RA SF. This finding is not consistent with most previous studies that reported significantly increased proportion of Th1 or Th17 in RA,^35^, ^36^, ^37^ which may be due to racial variation, disease heterogeneity and differences in drug treatment prior to patient sampling.

Numerous studies have indicated that Id2 serves a unique function in modulating various inflammation‐cancer diseases.^38^, ^39^, ^40^ However, its involvement in the pathogenesis of RA has not been clearly reported. Id2 can indirectly modulate target genes through its binding to other transcription proteins,^19^ allowing it to play diverse roles in different diseases. Most studies have indicated that Id2 effectively maintains the stemness of cancer cells, supporting their proliferation, migration and invasion.^41^, ^42^ Conversely, Id2 functions as a transcriptional repressor in the establishment of pathological Ca^2+^ channels in cardiomyocytes, exerting a protective role.^43^ In our study, Id2 expression was significantly increased in subsets of over‐activated pro‐inflammatory T cells in RA patients. Further analysis revealed a significant positive correlation between Id2 and IFN‐γ in both SF and PB of RA. The findings indicate that Id2 may participate in RA inflammation by driving cytokine expression in RA.

Id2 is a crucial regulator that determines the developmental fate of multiple immune cell lineages, including T cells.^44^ Animal studies have confirmed that Id2 preserves the capacity of the pro‐inflammatory Th1 subset to secrete IFN‐γ by directly inhibiting E‐protein binding to the T‐bet gene, thereby suppressing T‐bet expression during the immune effector phase following acute viral infection.^45^, ^46^ Unexpectedly, while Id2 expression was reduced in circulating CD4^+^ T cells in IBD, subsequent Th1 and Th17 differentiation increased.^25^ These studies suggest that, in addition to Id2's intrinsic indirect regulatory mode, the significant differences in the pathological mechanisms of various diseases may also contribute to its opposing effects. In our work, stimulation of T‐cell activation in vitro notably altered Id2 expression, Th1 and Th17 differentiation, and their respective cytokine secretions. To clarify the regulatory relationship, we used helichrysetin, a natural inhibitor of Id2, to inhibit Id2 function. The results showed a significant downregulation of IFN‐γ‐expressing T cells, as well as in the expression intensity of IFN‐γ. Although IL‐17‐expressing CD4^+^ T cells decreased, the level of IL‐17 expression did not change significantly. Similarly, the protein level of IL‐17 did not show a substantial elevation in naive CD4^+^ T cells that overexpressed Id2 during Th17 induction in vitro.^47^ This may be due to its low proportion, high plasticity or delayed protein translation.

The CIA experimental arthritis model has been extensively used to explore the pathophysiological mechanisms underlying RA and to ascertain pharmaceutical targets.^48^ Previous studies have reported that T‐cell‐specific Id2‐deficient mice, upon antigen stimulation, have an impaired effector CD4^+^ T‐cell population size and capacity to infiltrate the central nervous system (CNS), rendering them completely resistant to EAE.^23^ In our study, deleting Id2 resulted in a limited incidence and disease score of arthritis but did not completely eliminate the induction of CIA in Id2^fl/fl^ Cd4‐Cre^+^ mice. Additionally, Id2 deletion interfered with the generation of pro‐inflammatory T cells and cytokine secretion during the effector phase following immune stimulation but had no impact under normal conditions. Serve as a crucial element of the body's immune, CD4^+^ T cells exert a profound impact on the initiation and perpetuation of autoimmune disorders, including EAE and RA. However, unlike EAE, which heavily depends on CD4^+^ T‐cell invading the CNS for symptom progression, RA joint inflammation is driven by complex immune interactions among various cell types. This has been demonstrated in animal experiments where arthritis can still be induced despite the depletion of CD4^+^ T cells.^49^ These findings emphasise the heterogeneity and complexity of RA pathogenesis and the necessity to explore new potential regulatory targets, such as Id2.

Targeting the pro‐inflammatory cytokine TNF‐α and inhibiting T‐cell co‐stimulatory signal activation are potent strategies for treating RA. Nonetheless, certain patients remain refractory and demonstrate inadequate responses to various therapeutic agents.^17^ Clinical data have confirmed that treatment with ABA markedly downregulates the proportion of pro‐inflammatory T cells and cytokines secretion.^50^, ^51^ Consistent with these findings, we noted a substantial downregulation in the activation degree of circulating T cells (CD69) treated with ABA for 12 weeks, paralleled by a decline in the proportion of pro‐inflammatory T cells and the secretion of inflammatory cytokines. Additionally, our in vitro results showed that Id2 expression was remarkably reduced by ABA treatment. Therefore, targeted inhibition of Id2 may limit the population size of pro‐inflammatory T‐cell subsets in RA and serve as a novel therapeutic strategy against RA inflammation. However, it remains to be further studied whether targeted inhibition of Id2 or antagonism of its biological function can effectively control RA inflammation and its underlying molecular mechanisms.

This study has limitations. The primary results were derived from flow cytometry analysis, and CD3 was not incorporated into the gating strategy for T cells, which compromised the gating accuracy of CD8^+^ T cells. Moreover, MFI was utilised to measure the expression levels of cytokines and Id2; however, as an indirect evaluation index, it can only facilitate comparison and does not accurately reflect true expression levels. Moreover, the patients in this study were sourced from a single hospital and were limited in number. Given the heterogeneity of RA, the results necessitate expanding the sample size and considering the inclusion of a more diverse patient population, as well as extending the treatment follow‐up period to assess the longitudinal impact on Id2.

In conclusion, this study indicated that increased expression of Id2 in hyperactivated T‐cell subsets promotes IFN‐γ expression and contributes to RA inflammation. The data presented herewith offer a novel viewpoint from which to consider the aetiology of RA and may provide a potential target for effectively controlling joint inflammation in RA.

## AUTHOR CONTRIBUTIONS

All authors contributed to the conceptualisation and design of this study, as well as the analysis and discussion of experimental data. Ping Zhu, Zhaohui Zheng, and Junfeng Jia conceived the study and revised manuscript. Haoyang Sun, Jinlin Miao and Kui Zhang performed most of the experiments and drafted the manuscript. Peiyan Zhang, Haomiao Shen, Jiawei Wang, and Bei Zhang participated in the process of clinical data collection and analysis. All listed authors read and approved the final version for publication.

## CONFLICT OF INTEREST STATEMENT

The authors declare that the research was conducted without commercial or financial relationships that could be perceived as a potential conflict of interest.

## ETHICS STATEMENT

The Ethics Committee of Xijing Hospital approved the study involving human participants (KY20192006‐F‐1), while National Translational Science Center for Molecular Medicine approved the animal study (2021‐NTSCMM‐ID015).

## CONSENT TO PARTICIPATE

Informed consent was obtained from all individual participants included in the study.

## Supporting information



Supporting Information

## Data Availability

The data presented in this study are offered without reservation. Further inquiries may be addressed to the corresponding authors.
