# Supplementary material for: Id2 exacerbates the development of rheumatoid arthritis by increasing IFN‐γ production in CD4+ T cells
Source: Clin Transl Med. 2025 Mar 6;15(3):e70242. doi: 10.1002/ctm2.70242 (PMC11885165; doi:10.1002/ctm2.70242)
Supplement: Supplementary file 1 — Supporting Information [file CTM2-15-e70242-s001.docx]

**Supplementary material**

**Id2 exacerbates the development of rheumatoid arthritis by increasing IFN-γ production in CD4^+^ T cells**

**Haoyang Sun^a, 1^, Jinlin Miao^a, 1^,** **Kui Zhang^a, 1^, Peiyan Zhang^a^, Haomiao Shen^a^, Jiawei Wang^a^, Bei Zhang^a^, Junfeng Jia^a,^ *, Zhaohui Zheng****^a,^ *, Ping Zhu^a,^ ***

^a^ Department of Clinical Immunology, Xijing Hospital, and Department of Cell Biology of National Translational Science Center for Molecular Medicine, Fourth Military Medical University, Xi’an 710032, China.

*** Corresponding author**.

E-mail address:

[zhuping@fmmu.edu.cn](mailto:zhuping@fmmu.edu.cn) (P.Z.); [zhengzh@fmmu.edu.cn](mailto:zhengzh@fmmu.edu.cn) (Z.Z.); [junfeng2023512@163.com](mailto:junfeng2023512@163.com) (J J).

^1^ Authors contributing equally to this work.

**
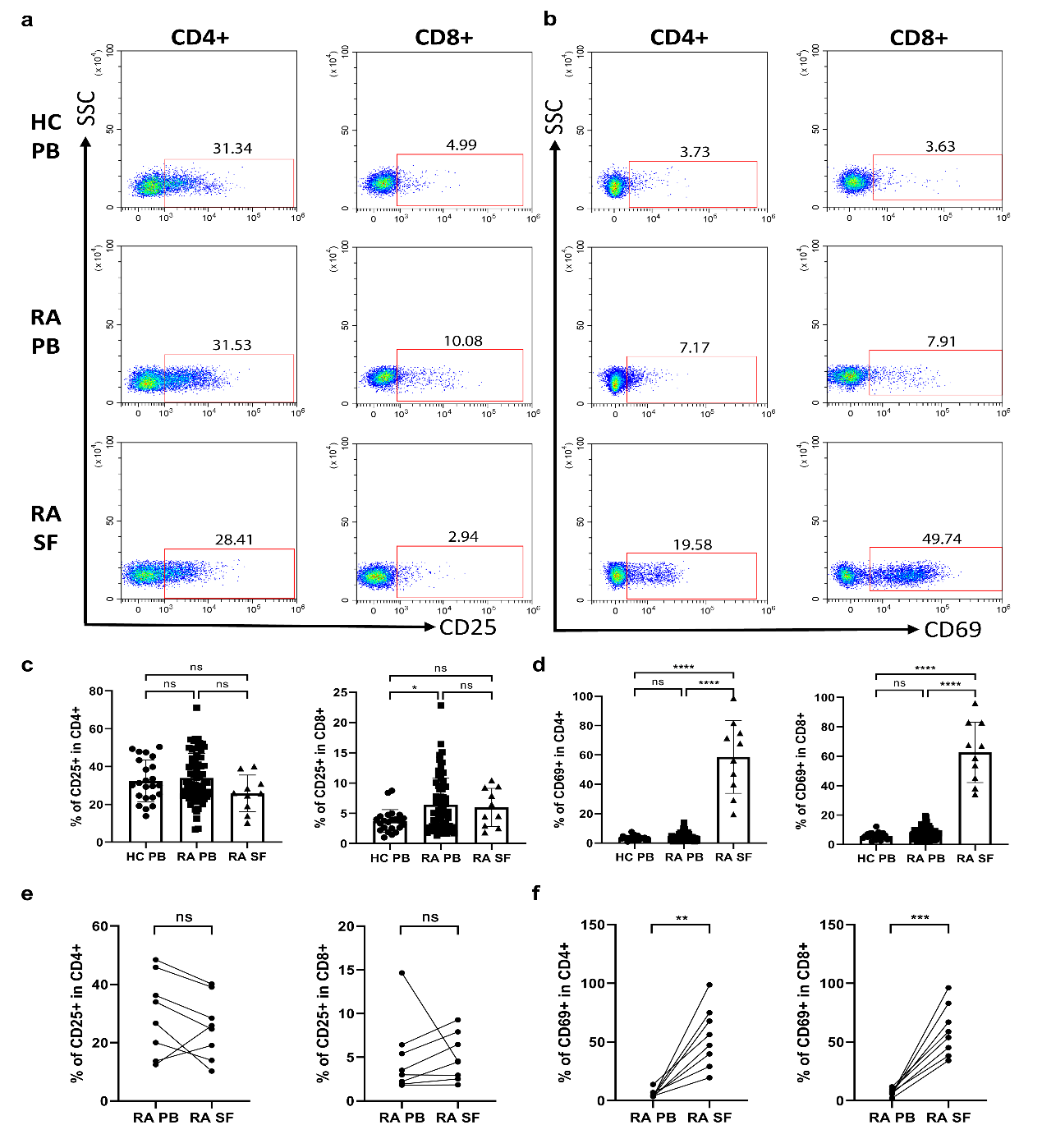
**

**Supplementary Figure 1. T-cell activation increased in synovial fluid of patients with active RA**

**(a, b)** Representative flow cytometric dot plots of CD25 and CD69 expression within CD4^+^ and CD8^+^ T cells in HC PB, RA PB, and RA SF. **(c, d)** Percentages of activation markers CD25 and CD69 on CD4^+^ and CD8^+^ T cells in HC PB, RA PB and RA SF. **(e, f)** Paired comparison of CD25 or CD69 expression levels in CD4^+^ and CD8^+^ T cells from matched RA PB and SF samples. Data are from 23 HCs, 69 RA patients, and 10 RA SF samples. Kruskal-Wallis H test followed by Dunn's test (c, d) and Paired Student's t-test (e, f) were used. ns: not significant; *: p < 0.05; **: p < 0.01; ***: p < 0.001; ****: p < 0.0001.

**
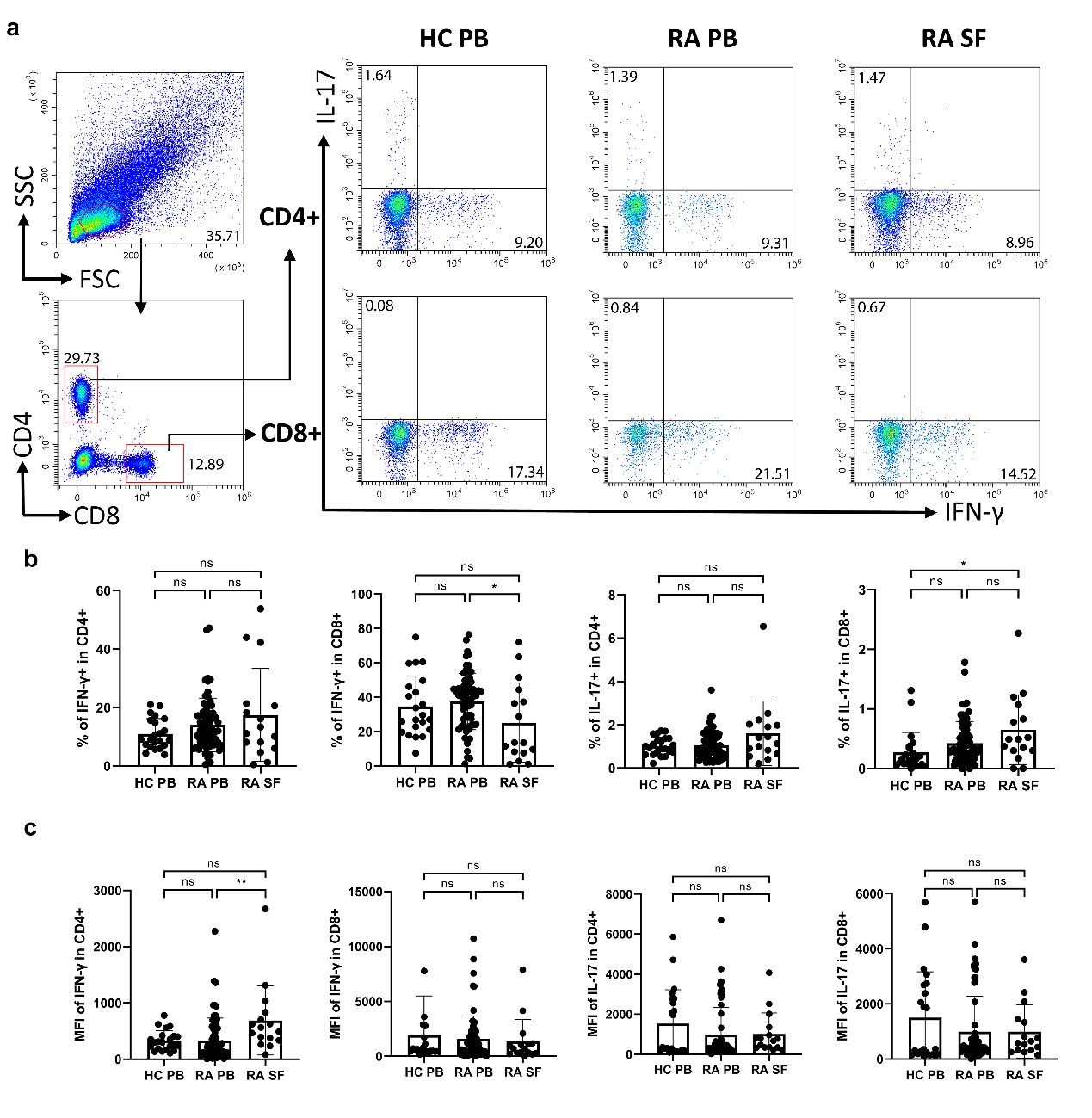
**

**Supplementary Figure 2. The increased expression of IFN-γ in T cells in synovial fluid of RA patients**

**(a)** Representative flow cytometric plots of IFN-γ and IL-17 expression in CD4^+^ and CD8^+^ T cells from HC PB, RA PB and RA SF. **(b)** Percentages of IFN-γ^+^ and IL-17^+^ in CD4^+^ and CD8^+^ T cells among HC PB, RA PB, and RA SF. **(c)** Expression of IFN-γ and IL-17 in CD4^+^ and CD8^+^ T cells from HC PB, RA PB, and RA SF. Data are from 22 HCs, 68 RA patients and 16 RA SF samples that were processed for stimulation prior to flow staining. Kruskal-Wallis H test followed by Dunn's test (b, c) was used. ns: not significant; *: p < 0.05; **: p < 0.01.


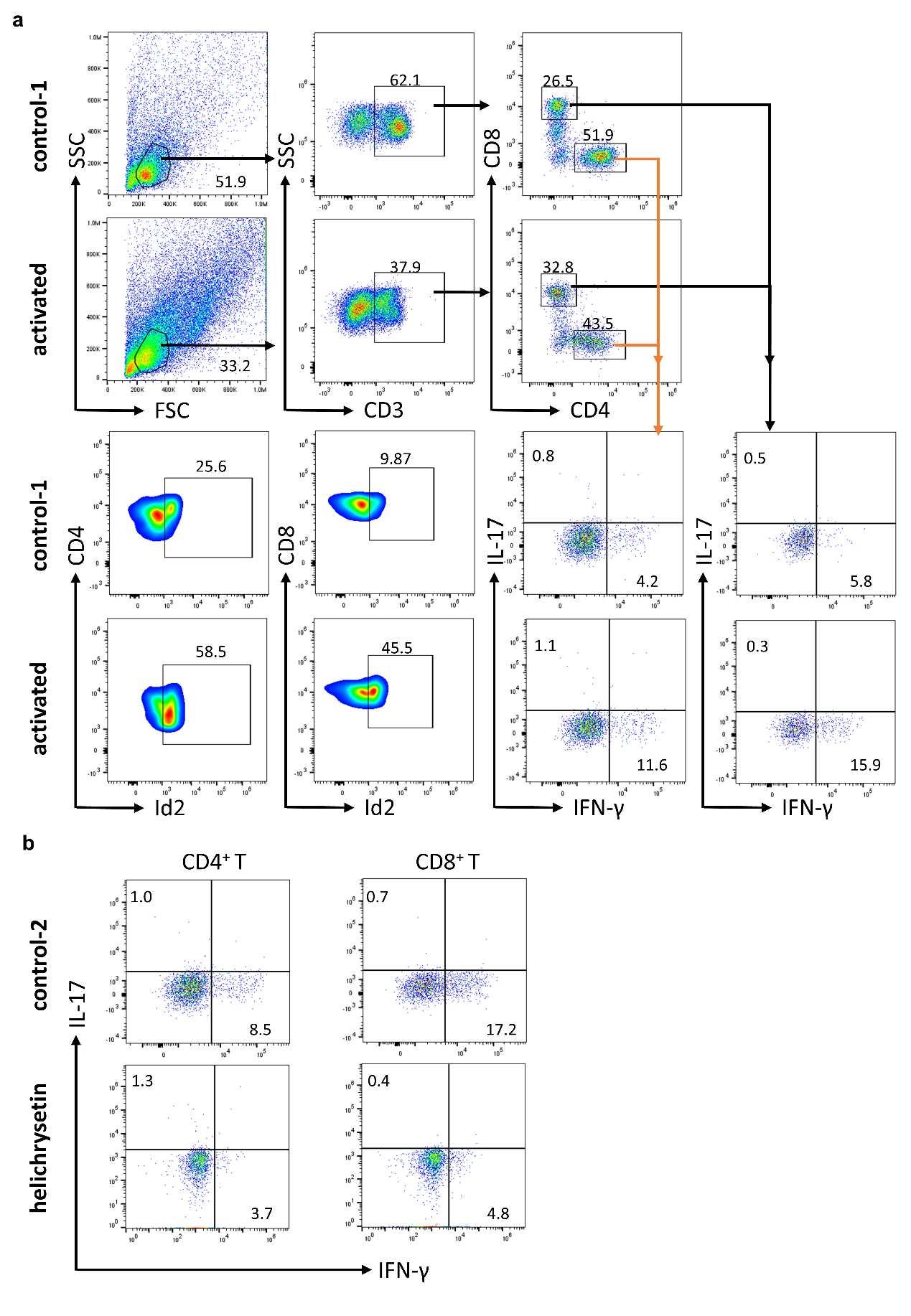


**Supplementary Figure 3. Flow gating strategies for *in vitro experiments***

**(a)** Representative dot plots illustrating the gating strategy for Id2-, IFN-γ-, and IL-17-expressing T cell subsets between inactivated (control-1) and activated samples*.* **(b)** Representative dot plots for IFN-γ- and IL-17-expressing T cell subsets between activated only (control-2) and activated + helichrysetin treated samples*.*


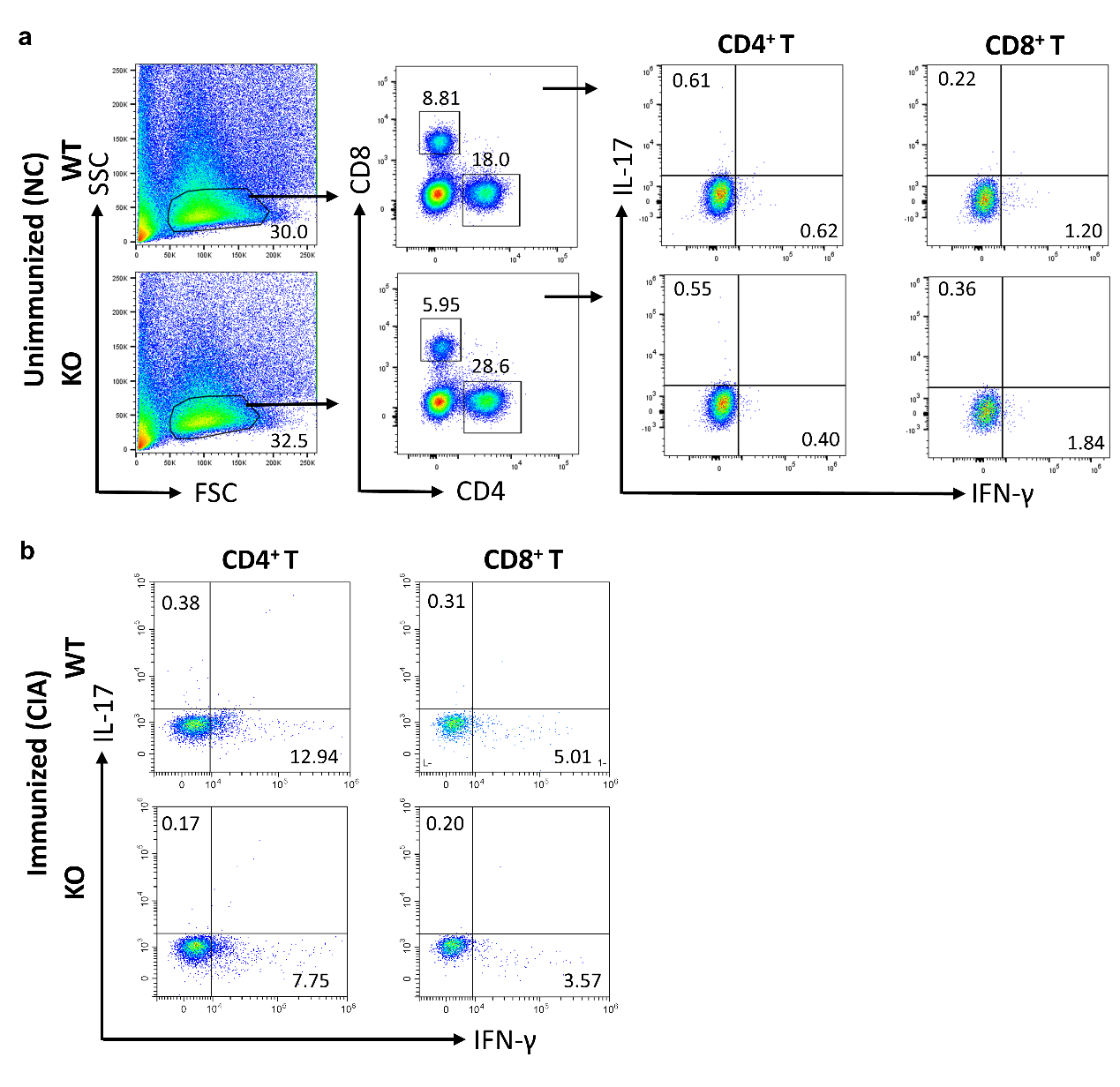


**Supplementary Figure 4.** **Flow gating strategies for animal experiments**

Representative dot plots illustrating the gating strategy for IFN-γ- and IL-17-expressing T cell subsets in unimmunized **(a)** and immunized **(b)** models.


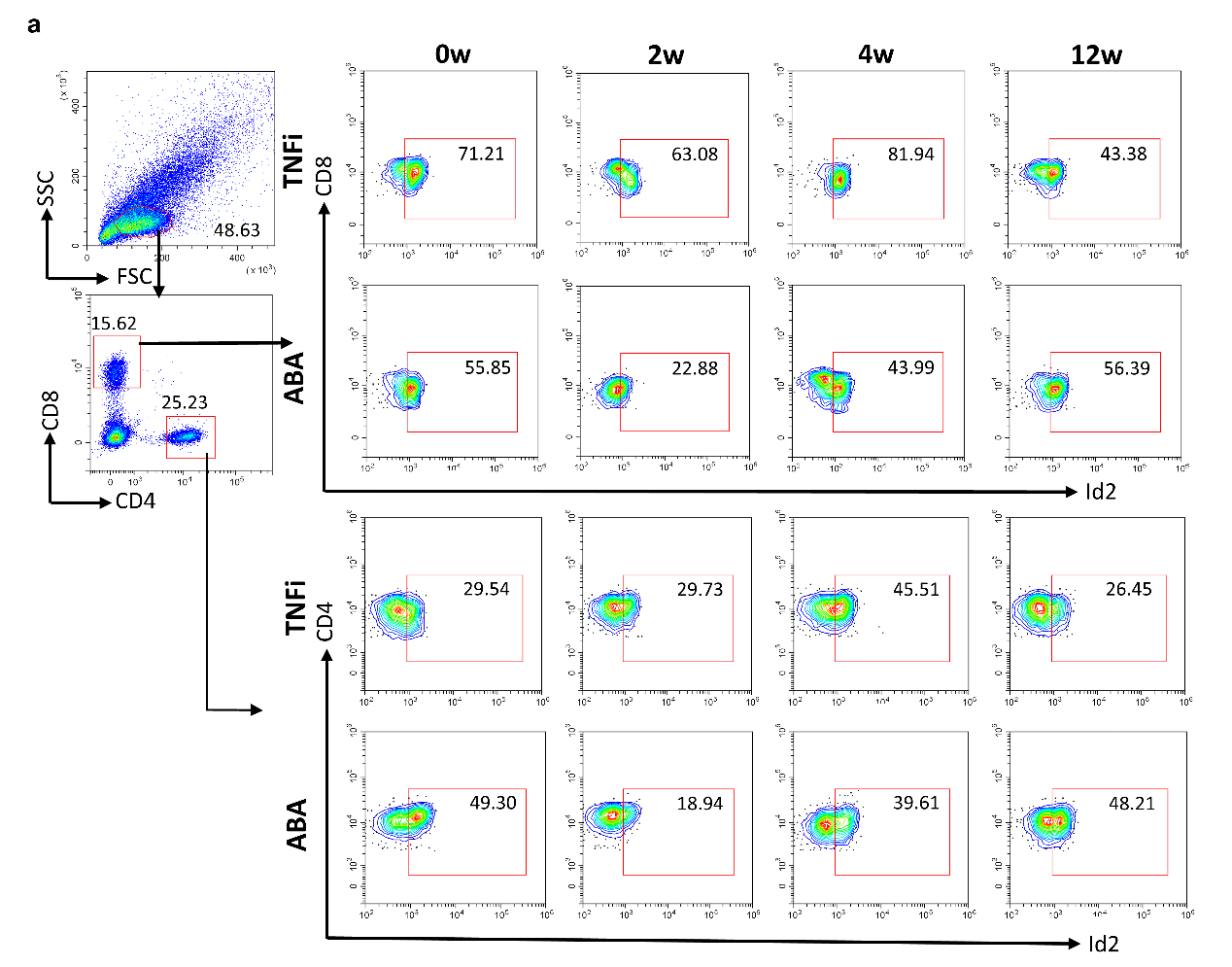


**Supplementary Figure 5.** **Flow gating strategies for Id2 during treatment of RA patients**

Representative dot plots of Id2^+^ cells at various time points between the TNFi and ABA groups of patients following treatment. TNFi: tumor necrosis factor inhibitors; ABA: abatacept.
